# Packet Flow Based Reinforcement Learning MAC Protocol for Underwater Acoustic Sensor Networks

**DOI:** 10.3390/s21072284

**Published:** 2021-03-24

**Authors:** Ibrahim B. Alhassan, Paul D. Mitchell

**Affiliations:** Department of Electronic Engineering, University of York, York YO10 5DD, UK; paul.mitchell@york.ac.uk

**Keywords:** MAC protocols, reinforcement learning, underwater acoustic sensor networks

## Abstract

Medium access control (MAC) is one of the key requirements in underwater acoustic sensor networks (UASNs). For a MAC protocol to provide its basic function of efficient sharing of channel access, the highly dynamic underwater environment demands MAC protocols to be adaptive as well. Q-learning is one of the promising techniques employed in intelligent MAC protocol solutions, however, due to the long propagation delay, the performance of this approach is severely limited by reliance on an explicit reward signal to function. In this paper, we propose a restructured and a modified two stage Q-learning process to extract an implicit reward signal for a novel MAC protocol: Packet flow ALOHA with Q-learning (ALOHA-QUPAF). Based on a simulated pipeline monitoring chain network, results show that the protocol outperforms both ALOHA-Q and framed ALOHA by at least 13% and 148% in all simulated scenarios, respectively.

## 1. Introduction

Medium access control (MAC) is one of the key requirements in underwater acoustic sensor networks (UASNs), garnering a major interest in the research community [1,2,3]. As an analogue of terrestrial sensor networks, UASNs are envisaged to enable a multitude of civilian and military applications [4,5,6]. To advance these applications, sensor nodes are being developed to be small/compact for easy transport, given that the environment is characteristically challenging to access. There is interest in new sensor nodes being energy efficient for longer deployments; as currently, there is no viable energy harvesting technology. Nodes should also be inexpensive to lower the overall cost, since UASNs are envisaged to be deployed to cover substantial marine areas and require a large number of devices. Employing acoustic waves in UASNs imposes some unique channel-centric constraints, such as: limited distance and frequency dependent capacity (bandwidth and data rate), long and variable propagation delay and high bit error rate (BER) on the design of UASNs [2,4,7]. As such, there is growing demand for efficient MAC solutions, especially adaptive MAC protocols for practical networks in the highly dynamic underwater environment.

Although preliminary studies on adopting existing MAC techniques/schemes from the vast body of work on terrestrial MAC protocols to underwater networks was largely found to be ineffective [1,8], the insight from the underlying principles remains useful. As a general guide, the network topology gives an insight into the appropriate category of MAC scheme to employ, with contention-free and contention based schemes better suited to centralised and decentralised topologies, respectively. Centralised topologies typically facilitate schedule creation and coordination from a central controlling node. Therefore, uncoordinated channel access becomes too contentious and less efficient. On the other hand, in a decentralised topology, such coordination is prohibitively challenging to implement, and the limited resources make contention-free protocols inefficient.

Code division multiple access (CDMA) and frequency division multiple access (FDMA) are promising contention-free schemes considered for UWASNs [9,10]. CDMA assigns unique binary codes to users (nodes) to spread the information signal, thereby offering the complete frequency band to nodes for simultaneous transmissions. Frequency hopping and direct sequence spread spectrum (FHSS and DSSS, respectively) are the standard modulations employed in this scheme. FDMA splits the channel into distinctive frequency bands and assigns them to different users. In this way, users can initiate concurrent transmissions without incurring collisions [5,10]. While the radio bandwidth (GHz) enables the implementation of these schemes with relative ease, in UANS, the available bandwidth is very limited (kHz).

Time division multiple access (TDMA) [11] creates schedules by splitting time into slots and is the most promising contention-free approach used in UASNs, because of its flexibility and potential to achieve true collision-free scheduling. Despite the challenges of synchronisation, some solutions leverage the long propagation delays for spatial reuse to improve performance. A gateway node in [12] creates a gap-free schedule and then requests packets from the transmitting nodes. Other solutions incorporate sleep cycles between activities to save energy [3]. The solution in [13] is for a central node to use an initialisation stage to gather network-wide information, which is then optimised using genetic and particle swarm algorithms to create a collision-free schedule. However, the lack of complete knowledge of the environment poses a major challenge for creating a lasting collision-free schedule.

Contention based MAC protocols such as ALOHA [14] and its variants offer low complexity and simplicity of implementation. The downside is that contention based protocols suffer low utilization and prohibitively large end-to-end delay at high loads due to the blind transmission strategy. The works in [15,16] integrated additional guard times between successive transmissions in order to reduce collisions, and reference [17] demonstrated receiver initiation (RI) to improve the performance. In RI, the receiver makes the first move of initiating the data transfer session by sending a request packet to the transmitter(s) (essentially polling). Since collisions occur at the receiver, the RI approach aims to eliminate the most common source of collision (transmit-receive collision). All these approaches add to the complexity, and the overheads incurred by the control packets limit the achievable utilisation.

A popular technique is to incorporate both contention based and contention-free components to form hybrid MAC protocols. This strategy improves performance by allowing networks/devices to switch to an optimum MAC scheme based on demand or traffic profiles. Variations in traffic were addressed in [18], where the protocol was pre-configured to assign capacity either by free assignment or on demand, and reference [19] balanced performance with two time slots in a frame, one slot for scheduled transmissions and the other for random access.

In the highly dynamic underwater environment, MAC protocols need to be adaptive to changing conditions as well. This is because previous assumptions used to create schedules may be outdated or sub-optimal due to changes in topology, traffic, node(s) failure(s) and/or addition(s). Reinforcement learning is a promising solution used in MAC protocols to provide adaptability and robustness in wireless sensor networks, such as ad-hoc emergency networks for disaster monitoring [20,21]. In such networks, intelligent MAC protocols will adapt to the changing topology or the environment. Instead of switching between MAC schemes, reinforcement learning is used to continually assess the network condition through feedback and appropriately responds with a view towards maintaining (as much as possible) a collision-free schedule.

In [21], we studied the use of ALOHA-Q [20] underwater. ALOHA-Q is a MAC protocol originally developed for terrestrial wireless sensor networks. It employs a Q-learning algorithm to incorporate intelligence into framed ALOHA. The frame is created with a predetermined number of periodic fixed time slots. Each slot is structured such that it accommodates a data packet, an ACK packet and their corresponding one hop propagation delays (Figure 2). Initially, nodes randomly select and transmit in any slot, but eventually, each node settles on a collision-free slot as the underlying Q-learning reward/punishment serves to reinforce successful slots. However, because the ACK serves as the critical signal for the reward/punish mechanism in the Q-learning algorithm, the overhead with respect to the slot size due to the long propagation delay severely constrains the effectiveness of the Q-learning strategy in terms of achievable utilization and end-to-end delay. In Section 3.2, we demonstrate the Q-learning update mechanism and how it is applied in the ALOHA-Q protocol.

The focus of this paper is to implement a robust, simple and computationally inexpensive MAC protocol that consistently and efficiently delivers the maximum channel utilisation in a monitoring chain UASN, such as an underwater pipeline. To achieve that, we were inspired by the research in [20,22,23,24]. For reference, Table 1 describes the terms/symbols used in this paper.

Our specific contributions are:

- To provide some background work on the feasibility of restructuring the slot size in a typical frame-based underwater MAC protocol to improve network performance.
- To propose a new slot structure with minimal overhead based on the relationship between packet transmission duration and the one hop propagation delay that is capable of achieving the theoretical channel utilization.
- To propose ALOHA-QUPAF, a novel dual-control intelligent approach to medium access control based on packet(s) flow in a linear chain network.

The rest of the paper is structured as follows. Section 2 introduces the frame based approach of the MAC protocol design and the network model. Section 3 presents the proposed slot size, the analytical modelling, and discusses the simulation results as compared to the theoretical results. It is followed by Section 4, our detailed dual-control intelligent MAC scheme, and the results obtained when applied to varying lengths of chain networks. Section 5 discusses the simulation results obtained of our proposed protocol. Finally, in Section 6 we draw conclusions.

## 2. Frame Based MAC Protocol

In this section, an overview is given of the fundamental operation of a baseline frame based random access protocol. With the aid of a simple network model, we analyse and identify the limitations of frame based scheduling (in terms of achievable channel utilization) with a random access scheme.

Framed ALOHA is one of the baseline protocols we compare against our proposed intelligent scheme. In contrast to slotted ALOHA, whereby time is divided into slots and nodes can only transmit at the beginning of each slot, a frame is used in framed ALOHA, which comprises a fixed number of contiguous slots $N_{s}$. In the framed ALOHA random access strategy, each node independently and randomly chooses one of the transmission slots at the beginning of each frame.

**Figure 1:**
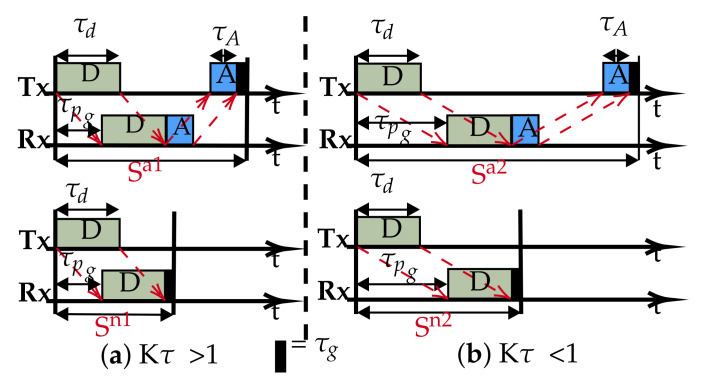
Typical slot structures: (**a**) Overlapping, transmission-reception occurs concurrently for the data packet. (**b**) Non-overlapping, data transmission completed before reception occurs.

Typically, a slot is structured such that it accommodates: a data packet of duration ($\tau_{d}$), an acknowledgement packet of duration ($\tau_{A}$ if required), the associated propagation delays of each packet ($\tau_{pg}$) and a small guard band ($\tau_{g}$): the band is essential to correct and guard against drifts in clock precision and synchronisation. The slot structure is shown in Figure 1, for cases with and without acknowledgements. Whereas, in radio networks, the overheads due to the wait period between successive data transmissions in a slot/frame can be of negligible length with respect to the packet duration, in an underwater acoustic channel however, the physics impose a long propagation delay, plus low capacity (bandwidth and therefore data rate), making the overheads significant, thus negatively impacting the channel utilization and end-to-end delay.

Defining the channel utilization (U) as the rate of delivering data at the designated sink node (Equation (1)), then, in frame/slot based protocols, the utilization is also a function of the number of slots ($N_{s}$) in the frame. For example, if a node is allowed to transmit *N* packets per frame, then the maximum effective utilization at the sink is going to be upper bounded at $N/N_{s}$. The value of $N_{s}$ is determined from the topology and interference population of the network. Setting $N_{s}$ inappropriately will negatively affect not just the utilisation, but potentially the stability of the MAC protocol as well. For example, in a star topology, $N_{s}$ is equal to the number of transmitting nodes ($N_{n}$); as each node should have a unique transmitting slot, setting $N_{s}>N_{n}$ adds extra un-utilised slot(s), and $N_{s}<N_{n}$ will cause contention as some nodes will not exclusively own a slot. Therefore, for a particular topology and interference model, there is an optimum $N_{s}$ ($N_{opt}$) [20]. Erlang [25] is a dimensionless unit that represents continuous channel usage (for example 0E = zero channel activity, 0.5E = half channel activity and 1E = full channel usage).

(1) $$U_{normalised}\left(Erlang\right)=\frac{N\times \tau_{d}}{N_{s}\times S}$$

therefore, the optimum utilization is:

(2) $$U_{normalised}\left(Erlang\right)=\frac{N\times \tau_{d}}{N_{opt}\times S}$$

where *S*, $\tau_{d}$ denote the slot duration and packet duration in seconds respectively.

One of the consequences of having low capacity is the long transmission duration, which presents two situations for a given transmitter and receiver pair: the transmission duration is either greater than or less than the propagation delay between the nodes. Following [26], if we introduce the parameter $K_{\tau }$ (Equation (3)), then the resulting slot structure can have either of two sets of transmission-reception patterns: overlapping and non-overlapping based on the value of Kτ, as shown in Figure 1.

(3) $$K\tau =\frac{\tau_{d}}{\tau_{pg}}$$

$S^{a1}$ and $S^{a2}$ represent the slots’ length with ACK and are typically used by slotted protocols employing an ACK signal such as ALOHA-Q. Similarly, $S^{n1}$ and $S^{n2}$ are the slots without ACK as used in framed ALOHA and TDMA. Equations (4) and (5) are used to calculate the slot sizes.

(4) $$S^{a}=\tau_{d}+\tau_{A}+2\;\tau_{pg}+\tau_{g}$$

(5) $$S^{n}=\tau_{d}+\tau_{pg}+\tau_{g}$$

In this slotted concept, nodes are allowed to transmit only one packet per frame (i.e., N = 1), and the expression of maximum utilisation (U) can be simplified to the ratio of packet duration-to-frame size (Equation (6)). We can combine Equations (2) and (6) to calculate the expression of the utilisation below:

(6) $$U=\left\{\begin{array}{cc} \frac{\tau_{d}}{{{N_{o}}_{p}}_{t}\;\left(\;\tau_{d}+2\tau_{pg}+\tau_{A}+\tau_{g}\;\right)}, & S^{a}\\ \frac{\tau_{d}}{{{N_{o}}_{p}}_{t}\;\left(\tau_{d}+\tau_{pg}+\tau_{g}\;\right)}, & S^{n} \end{array}\right.$$

As $\tau_{d},\tau_{pg}>>\tau_{A},\tau_{g}$, Equation (6) approximates to:

(7) $$U\approx \left\{\begin{array}{cc} \frac{\tau_{d}}{{{N_{o}}_{p}}_{t}\;\left(\;\tau_{d}+2\tau_{pg}\;\right)}, & S^{a}\\ \frac{\tau_{d}}{{{N_{o}}_{p}}_{t}\;\left(\tau_{d}+\tau_{pg}\;\right)}, & S^{n} \end{array}\right.$$

From Equation (7), it can be seen that, since $\tau_{d}$ and $\tau_{pg}$ dominate, the value of Kτ will guide us on how to improve channel utilisation by restructuring the slot size. For Kτ>1, we are constrained with respect to any change to the slot size. Any reduction will create overlapping slot reception that will effectively render the slotting meaningless, as demonstrated with the downgrade of slotted ALOHA to pure ALOHA underwater [26].

**Figure 2:**
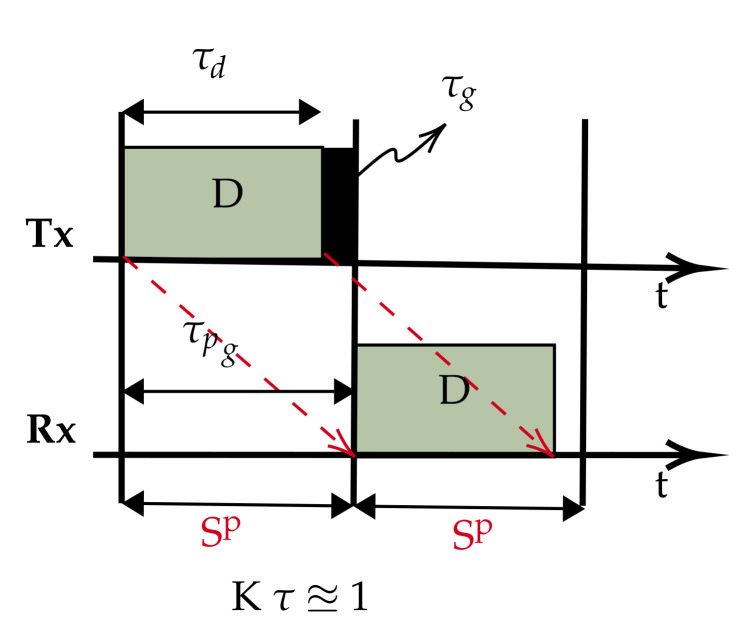
Proposed slot structure.

In most UASNs applications, the propagation delay is longer than the transmission duration because of sparse connectivity. Therefore, Kτ<1 best describes such scenarios. We propose the slot structure in Figure 2. The slot size is now reduced to approximate the propagation delay ($S\approx \tau_{pg}$), which is possible since with Kτ<1, the data packet can be safely accommodated in $\tau_{pg}$. This simple slot structure aims to reduce and fill the otherwise wide gap in the conventional slots with useful data (compared to Figure 1). Therefore, for a given chain UASN, designed with nodes separated by a *d*m transmission range, we demonstrate that there are advantages to the performance improvements of using our slot structure; for example, the peculiar characteristic of the underwater communication channel in terms of its distance dependent capacity, that is the acoustic transmission bandwidth and data rates decrease with increasing transmission distance [27]. As such, instead of a few hops transmitting over longer ranges (requiring high power) with low capacity, we can potentially achieve higher capacity transmissions with additional hops added to route data over shorter ranges (low power). To investigate the achievable utilisation, the slot structure shown in Figure 2 is based on Kτ≈1: a special case of Kτ<1. This is purely to limit the overhead in the slot, as increasing the slot size beyond $\tau_{pg}$ negatively affects the utilisation according to Equation (6).

### Scenario and Network Model

**Figure 3:**
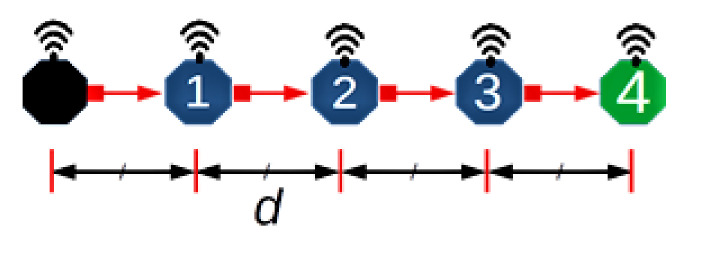
An example scenario.

Consider a scenario comprising quasi-stationary equally spaced nodes in an N hop underwater network chain topology, with data delivered along the chain from one end to the other. Figure 3 depicts an example of such a network with N = 4 and hop distance *d*. This topology is representative of pipeline monitoring. As such, during the reporting cycle, the network can be considered loaded to capacity; accordingly, this work is primarily concerned with the achievable utilisation. To aid the analysis, the following assumptions are made:

1. All nodes are homogeneous and communicate over a single channel, half-duplex mode.
2. The collision model (non-capture) is used, i.e., if two or more packets overlap at the receiver, they are discarded.
3. Nodes are globally synchronised, an assumption commonly employed to simplify analysis and applicable to quasi-stationary nodes synchronised before deployment.
4. The interference range (Ifx) is twice the reception range (Rx); this model is typically employed for chain networks as an illustrative model to incorporate the effect of interference from nodes that are two hops away.
5. A source node has saturated traffic, i.e., always has a packet to send, to provide the maximum monitoring rate based on the transmission opportunities offered by the MAC layer. Similar research papers are concerned with achievable utilization [23,28,29].
6. All source/relay nodes can only transmit one packet per frame, a consequence of Assumption (4) yielding a frame consisting of four slots [20], as only one of four connected nodes can transmit successfully at a given time.

We re-write Equation (7) of $S^{n}$ to get the new utilisation for the proposed slot structure:

(8) $$U_{normalised}\left(Erlang\right)=\frac{\tau_{d}}{{{N_{o}}_{p}}_{t}\times \tau_{pg}}$$

and in terms of $K_{\tau }$, it becomes:

(9) $$U_{normalised}\left(Erlang\right)=\frac{K_{\tau }}{{{N_{o}}_{p}}_{t}}$$

In summary, while the traditional slot structure that incorporates the propagation delay and/or ACK packet within the constraints of the available channel resources, we show that with Kτ<1, the propagation delay is sufficient to accommodate the data packet, then it is possible for the slot size to be effectively reduced and restructured (by at least 50% of the cases in the Kτ<1 regime), and as long as a protocol does not require an ACK packet, there is a potential for a dramatic improvement in performance (Equation (9) vs. Equation (7)).

## 3. Model Analysis

To analyse the network with the proposed slot structure (Figure 2), we consider a baseline scheme whereby each node initialises by randomly choosing a transmission slot. The purpose of considering this scheme is first to demonstrate the inefficiency of a random access scheme by analysing the distribution of the achievable channel utilization, second to investigate the feasibility of applying intelligent techniques to the model that could lead to a significant performance improvement and, finally, to evaluate the efficacy of the proposed slot structure coupled with the intelligent techniques relative to similar intelligent approaches and random access baseline schemes.

**Figure A2:**
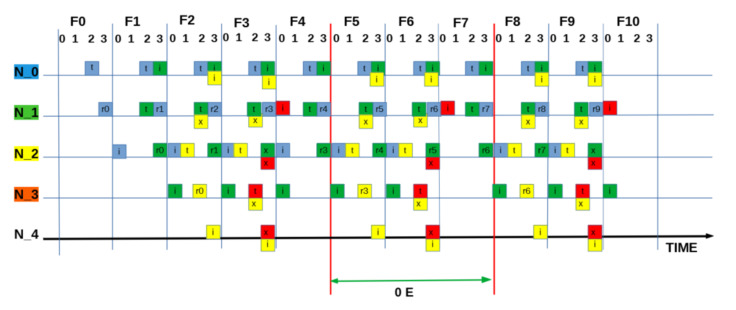
SEQUENCE:[ 2 2 1 2 ]: “Worst” measured utilisation based on zero packets being delivered = 0.0 E.

**Figure A3:**
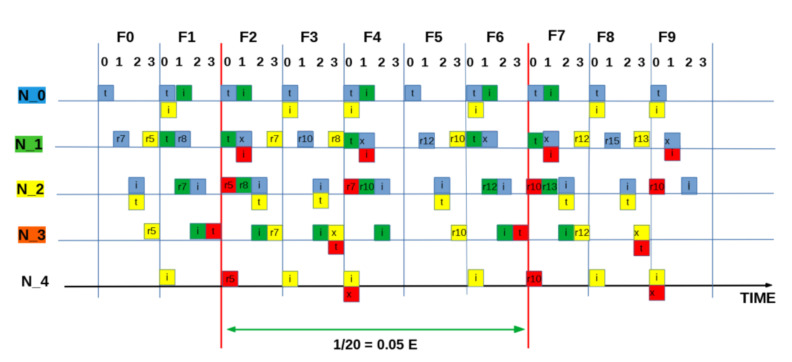
SEQUENCE:[ 0 0 2 3 ]: “Intermediate” measured utilisation based on one packet in five frames (20 slots) = 0.05 E.

**Figure A4:**
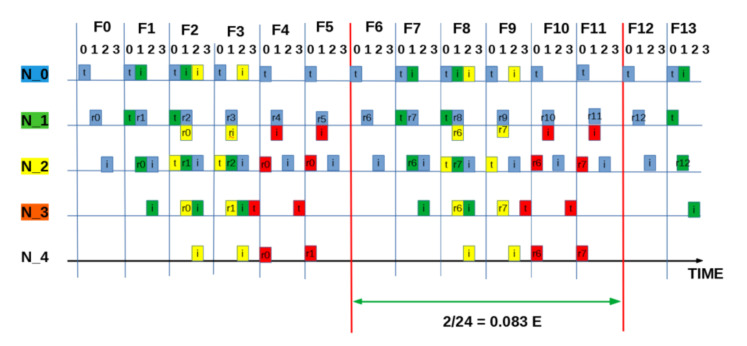
SEQUENCE:[ 0 0 0 3]: “Intermediate” measured utilisation based on two packets in six frames (24 slots) = 0.083 E.

**Figure A5:**
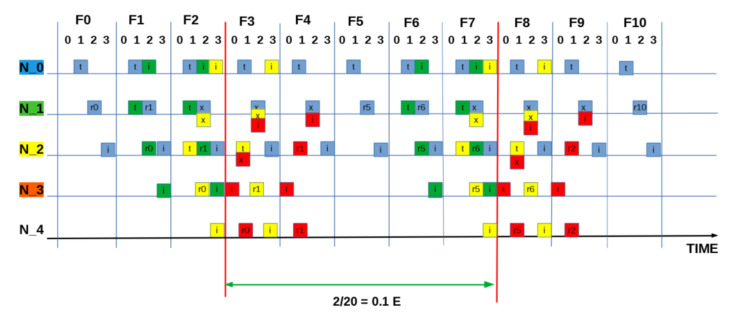
SEQUENCE:[ 1 1 1 0 ]: “Intermediate” measured utilisation based on two packets in five frames (20 slots) = 0.1 E.

**Figure A6:**
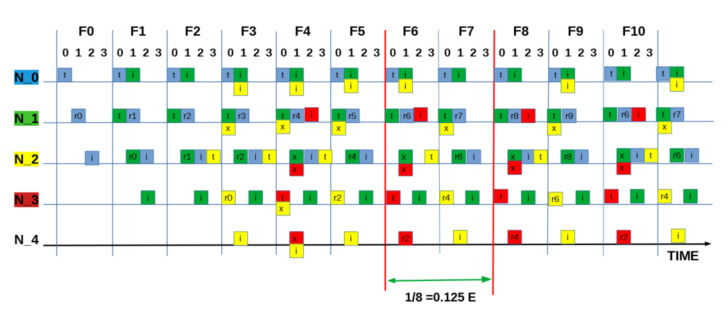
SEQUENCE:[ 0 0 3 0 ]: “Half” measured utilisation based on one packet every two frames (8 slots) = 0.125 E.

**Figure A7:**
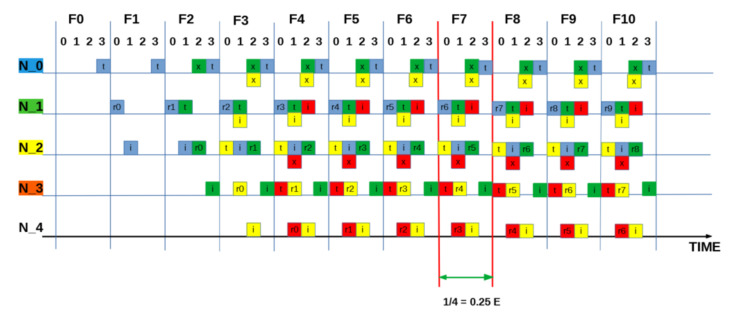
SEQUENCE:[ 0 2 1 1 ]: “Best” measured utilisation is one packet in every frames (4 slots) = 0.25 E.

To build the frame, we start with the optimal number of slots per frame $N_{opt}$. In a linear chain network (such as Figure 3 and longer,) $N_{opt}$ is four as computed according to the two hop interference model [20]. This is because in a linear topology with two hop interference model, technically only one in four nodes can successfully transmit at a given time. Similarly, for one hop and three hop interference models, one in three and one in five nodes can transmit successfully [20,23]. Therefore, for a distributed MAC protocol, such as framed ALOHA employed in this setup, each node is free to chose any of the available four slots in the frame, resulting in $4^{4}=256$ ways for nodes to independently select and occupy transmission slots. Table 2 lists the range of the 256 possible slot combinations in a four column array of 64 unique patterns, with each column vector signifying the transmission slot pattern from Node 0 to Node 3. That is, the vector [0000] denotes all nodes selecting and occupying Slot 0; likewise, slot sequence [2210] signifies both Nodes 0 and 1 choosing Slot 2, while Nodes 2 and 3 choose Slot 1 and Slot 0, respectively. Pictorial timing depictions (see Appendix A) are employed to observe and obtain the theoretical bounds of the scheme in terms of channel utilisation. The diagrammatic method provides a visual intuition of our core idea. In Appendix A, six examples (Figure A2, Figure A3, Figure A4, Figure A5, Figure A6 and Figure A7) are provided to illustrate the process. For each pattern, N_0 is the source node; it generates and transmits data in every frame to N_1, which forwards the packet (if successfully received) to N_2 in the next frame, and so on. Overall, individual packets are traced frame-by-frame as they traverse the network from source to sink (N_0toN_4). The final utilisation is measured when an overall periodic pattern emerges at the sink node (vertical red lines in each example figure; refer to Appendix A).

### 3.1. Results

In order to empirically evaluate the performance of the above random access scheme, we ran a simulation on a network of five nodes (Figure 3) configured with the proposed slot structure analysed in Section 3. Each node is pre-configured to run a MAC protocol that randomly selects and maintains a transmission slot at the beginning of each simulation run. It should be noted that in this simulation, since $K_{\tau }\approx 1$, the transmission delay and propagation delay are abstracted to 1:1 for the best results.

**Figure 4:**
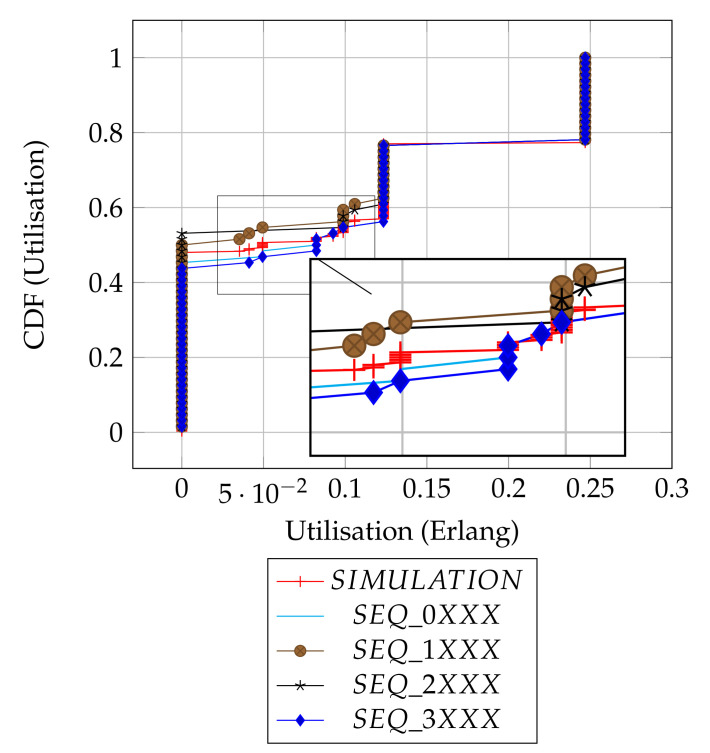
Distributions’ comparison.

Figure 4 shows and compares the utilisation results from both the analytical distributions of the slot patterns and the simulations. Overall, there are three dominant utilisation levels and some spurious intermediate levels, as summarised in Table 3. The summary provides individual proportions of levels in each plot, and the overall column is the contribution of each sequence in the combined set of 256 slots.

Depending on the chosen slot by the source node, transmissions could be initiated from either the frame edge (Slots 0 or 3) or mid-frame (Slots 1 or 2), and to some degree, the results show how the position of a chosen slot affects the utilisation. As shown in the result summary (Table 3), there is a subtle, but clear advantage in performance when the source node initiates transmissions with emerging slot patterns at frame edges (i.e., SEQ_0XXX, SEQ_3XXX) relative to the mid frames (i.e., SEQ_1XXX, SEQ_2XXX) or there is at least an 8% better chance of getting a packet received at the sink node when the source node transmits at the edges of a frame as compared to when source node uses mid frame (in terms of the worst case utilisation levels).

Intuitively, the distribution of the utilisation of the patterns can be assumed to be similar, since it can be demonstrated that each column sequence can be translated to another corresponding sequence in the remainder of the columns (Table 2). However, due to the transmission strategy of the protocol of scheduling packet transmission at the beginning of each frame, the simple slot structure guarantees that packets transmitted at $slot_{i}$ be received at $slot_{i+1}$. This means sequence translations will result in packet reception/interference across frames, consequently causing the distribution of the utilization outcomes to vary. For example, consider the corresponding slot selection sequences: [ 0 0 3 0 ], [ 1 1 0 1 ], [ 2 2 1 2 ] and [ 3 3 2 3 ]. [ 0 0 3 0 ] and [ 3 3 2 3 ] both have cross-frame receptions and have a similar utilization of 0.125 Erlangs (Figure A7). In contrast, [ 1 1 0 1 ] and [ 2 2 1 2 ] have no cross-frame reception and yield 0 Erlangs (Appendix A: Figure A2). Only 60 out of the total 256 slot sequences yield the maximum utilization level as a whole and remain immune to the slot sequence translations because they are perfectly collision-free. In Figure A2, Figure A3, Figure A4, Figure A5, Figure A6 and Figure A7 (Appendix A), we show how we computed six of the ten prominent utilisation levels for brevity.

The simulation results are in agreement with our analytical results, as they show that no data is delivered 48% of the time. This corresponds to the average of the possible 43–53% worst cases in the given original slot patterns, as expected. Most importantly, the simulation result confirms that the full channel utilization is achievable with the exact proportion of 23%. Finally, the simulation result shows the average performance of the random slot selection protocol and will serve as a baseline with which to demonstrate the merit of slot based learning in the new protocol ALOHA-QUPAF.

### 3.2. Q-Learning

This section demonstrates the underlying Q-learning update procedure based on stateless Q-learning [30]. Following the standard Q-learning framework, an agent learns how to behave in an unknown environment by interaction with the environment. The agent perceives and changes the state of the environment by taking an action and receives a response from the environment, which indicates the quality of the action taken in the form of a reward/punish signal. This process is Markovian, and it is modelled as an MDP [30,31,32]. To develop a MAC protocol, this is translated to a node taking the action of transmitting the data packet, and the successful/unsuccessful reception of an ACK packet represents the reward/punish signal. Each node is given a vector of Q-values (Q-table), and each Q-value is in turn assigned to one slot in the frame (Section 1). At the beginning of each frame, a node will scan the Q-table and select the slot with the highest Q-value to schedule transmission in that slot. Successful transmissions are rewarded and unsuccessful transmissions punished based on the reception or otherwise of an ACK packet and updating the Q-value of the transmission slot using Equation (10).

(10) $$Q\left[S_{t}\right]\leftarrow Q\left[S_{t}\right]+\alpha \left(\psi -Q\left[S_{t}\right]\right)$$

where $Q[S_{t}]$, α and ψ denote the Q-value of the current slot, the learning rate (0.1) and the reward/punish signal (±1).

Table 4 illustrates an example of the Q-learning as implemented in ALOHA-Q. Consider an initial situation (Frame 0) whereby a node i with data to send randomly chooses Slot 2 (because all slots have equal Q-values) at the beginning of a frame to schedule transmission and the transmission was unsuccessful.

- The new Q-value of Slot 2 becomes;
  Q[2]←0+0.1(−1−0) ; [−0.1]
- In the next frame, Slot 2 has the lowest Q-value and is not considered, and the node again chooses Slot 1 randomly (among Slots 0, 1 and 3). Following a successful ACK reception, the new Q-value of Slot 1 is updated.
  Q[1]←0+0.1(+1−0) ; [0.1]
- For Frame 2, the node chooses Slot 1 as it has the highest Q-value (0.1) and sends data; with successful ACK reception, the Q-value is updated accordingly.
  Q[1]←0.1+0.1(+1−0.1) ; [0.19]

The table gives the Q-values up to twenty frames assuming Slot 1 continues to be successful. This simple, yet effective recursive Q-learning update bootstraps the trial-and-error mechanism to a robust collision-free schedule as each node will eventually and independently occupy a unique transmission slot.

However, as previously stated, while the ACK signal is crucial to the Q-value update operation, it puts an additional burden on the scarce network resources underwater: reducing utilisation due to overheads and increased delay due to the ACK signal wait times. Our goal is to implement a novel Q-learning approach that maintains the level of intelligence without this explicit ACK signal, thereby maximising the channel utilisation and improving end-to-end delay.

## 4. Underwater Packet Flow ALOHA-Q: ALOHA-QUPAF

The proposed slot structures in Figure 2 pose a critical question: how do we apply a simple reinforcement learning algorithm to ultimately achieve collision-free scheduling without an ACK packet? In this section, we present a two stage solution using a reformulated Q-learning coupled with a simple stochastic averaging expression [33], the harmonised stages are described in Algorithm 1. We demonstrate the efficacy of our dual-mode learning approach in improving performance in a chain network as introduced in Section 2.

### 4.1. Protocol Design

In order to achieve the goal of realising a collision-free schedule without an explicit ACK signal, we modified the Q-value update process (Section 3.2) while maintaining the remaining protocol settings and assumptions (Section 2 and Section 3.2). Specifically, at the beginning of each frame, a relay node chooses the slot with the highest Q-value (if more than one slot has the highest Q-values, one is chosen at random) to forward a received packet on to the next hop. In the case of the source node, it initialises by randomly selecting and maintaining a constant slot for transmission. This is because we employ a Q-learning process that utilises packet receptions to update and reinforce transmission slot selection. Our solution involves a two stage approach based on the following intuitions:

1. In a network with half-duplex nodes, they cannot transmit and receive at the same time (slot); therefore, we employ Q-learning to isolate all reception slots by punishing those slots to lower their Q-values. As such, when a node scans the Q-table, reception slots will have low Q-values and are unlikely to be selected for transmission.
2. A continuous flow of packets over the chain is expected in saturated traffic with a healthy channel. Thus, a relay/sink expects a new packet(s) in every frame after receiving the first packet, and a packet collision is inferred whenever that stream of packets gets disrupted. To exploit this realisation, every time a relay node transmits a packet, it rewards the chosen transmission slot (positively updates the slot’s Q-value) if and only if a new packet is received afterwards.

We denote the two stages in the dual mode control as slot selection and flow harmony, and a detailed description of the process is given below:

- **Slot selection:** This is implemented by Q-learning to eliminate the reception slot(s). When a source node generates a packet and transmits, upon receiving the packet, the receiver (relay node) will record the reception slot (rx_s) and update the Q-value of the slot according to (Equation (10)). Specifically, each slot in a frame is mapped to a value in the vector of Q-values (Q[ns]), and the Q-values are initialised with a uniform random number less than one, whereby for each reception, the node computes rx_s and updates Q[rx_s] accordingly with ψ=−1. Consequently, this continual negative reinforcement of reception slots isolates those slots, and the slot(s) with the highest Q-value(s) signifies a probable collision-free slot at the local level, therefore a good candidate(s) slot(s) for transmission. For a relay node, at the beginning of each frame, if a node has a packet(s) in its queue, it will schedule a packet transmission in a slot with the maximum Q-value; however, if more than one slot shares the maximum Q-value, one will be chosen at random from amongst them. Whilst the Q-value of the reception slot is always punished following any reception, the Q-value of the transmission slot is only updated after every transmission. If there is a subsequent packet reception, the transmission slot is rewarded (ψ=1), otherwise it is punished (ψ=−1). However, since this scheme lacks a definitive feedback signal based on this node action(s) of transmissions, the success of any transmission in the chosen slot is uncertain. This is because, unless if the packet flow is network wide, a continuous transmission and reception by a relay node does not mean that a given node’s transmissions are not interfering with some other transmissions especially for the downstream links. Therefore, to avoid nodes from getting stuck in local minima, a control mechanism has to be devised to regulate the Q-values especially of the transmission slot.
- **Flow harmony:** Although we devise a means to obtain feedback from the environment (reward/punishment), the node cannot directly link these signals to its own action(s); hence, at any given time during the network run, we only have a partial observation of the channel condition; this type of process is best modelled as a partially observable Markov decision process (POMDP) [34,35]. This is because, instead of certainty in the network wide flow, the packet flow experienced by each node gives us a partial observation on the channel at the local level. The POMDP framework enables us to model the local observations by agents to generate a probability distribution of a belief state (in our case, settled or unsettled flow). The network can be in either stable or unstable packet flow states, and we therefore designate two belief states accordingly. We employ a simple heuristic strategy based on the stochastic averaging [36], whereby each node independently tracks its overall local packet flow in a given window, which we then translate as the distribution of the belief state. The distribution of the belief states is computed with Equation (11). For each reception in a frame, $fl_{\tau }$ is updated by λ steps at the tracking rate γ. While the expression monotonically approaches one, it is continually windowed every ($W_{n}$) frames and compared to a fixed threshold (thresh). Based on our simulation experiment, ideally, $fl_{\tau }$ will reach 98% by the 20th frame; hence, we heuristically set ($W_{n}=20$) to check for $fl_{\tau }$ with a tolerance of thresh=95%, which should be achieved at ($W_{n}=14$).
  If we designate the belief states S1 and S2 respectively as the initial state (both Q-values and $fl_{\tau }$ reset; the network is assumed to have no stable flow during learning) and the flow harmony state, S1 is decided when the averaging function exceeds the threshold, which indicates that flow harmony has been achieved at least in the node’s local interference group, otherwise the node resets to S2. In essence, every node has a window of 20 frames to isolate incoming reception slots and settle on a transmission slot. Whenever a particular node(s) fails to settle and join the flow, the reset will make the node switch to another slot and potentially notify other nodes in the neighbourhood as well.
  (11) $$fl_{\tau }\leftarrow \left(1-\gamma \right)fl_{\tau }+\lambda$$
  where $fl_{\tau }$, γ and λ denote the flow averaging, the learning/tracking rate and the increment scale, respectively.

By using this two stage solution, ALOHA-QUPAF unlike ALOHA-Q effectively isolates both reception slots from the transmission slots and finds an implicit way of getting the feedback signal of the node’s action based on the individual nodes experiencing successful reception of a continuous stream of packets. Furthermore, it differs from framed ALOHA, since it can intelligently create and maintain a robust collision-free schedule. The complete ALOHA-QUPAF algorithm is given below.
 **Algorithm 1:** ALOHA-QUPAF algorithm. 
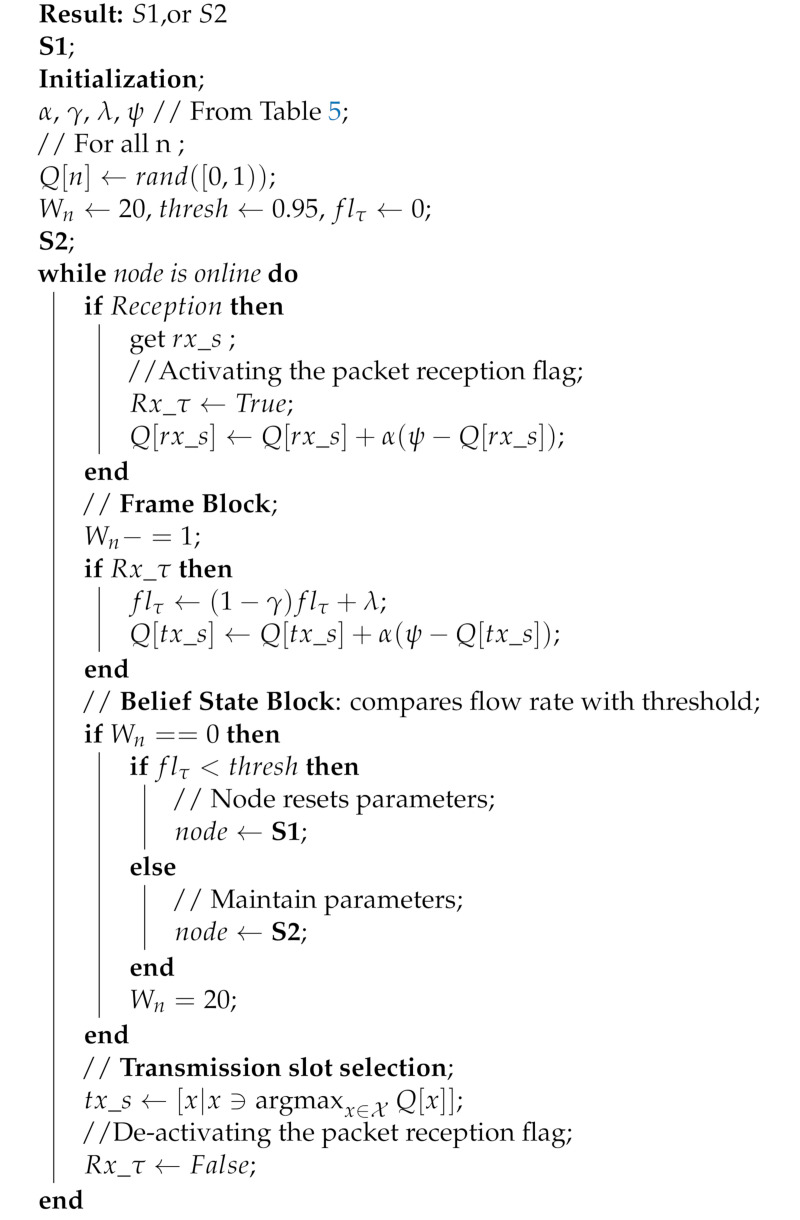


### 4.2. Results

Since the focus of this work is principally to improve performance in terms of channel utilization measured at the sink, ALOHA-QUPAF is compared to a state-of-the-art ALOHA-Q, which employs a similar Q-learning technique, and a baseline framed ALOHA scheme in terms of the normalised utilization. We simulated networks of varying hop lengths with the protocols configured with respect to the structures in Figure 2. For a fair comparison, as our proposed slot structure is constrained to Kτ>1, we only compare ALOHA-QUPAF with the other protocols in the Kτ>1 regime. The network was simulated in the Riverbed Modeler (formerly OPNET) environment, and the setup used the parameters given in Table 5, which were based on a modem developed by Newcastle University [37]. In all cases, the network was simulated for 15,000 frames, with a single saturated source at one end of the network and a sink at the other end. In terms of result collection, due to the continuous nature of the learning of the ALOHA-QUPAF algorithm, the results were collected from the beginning of the simulation.

## 5. Discussion

**Figure 5:**
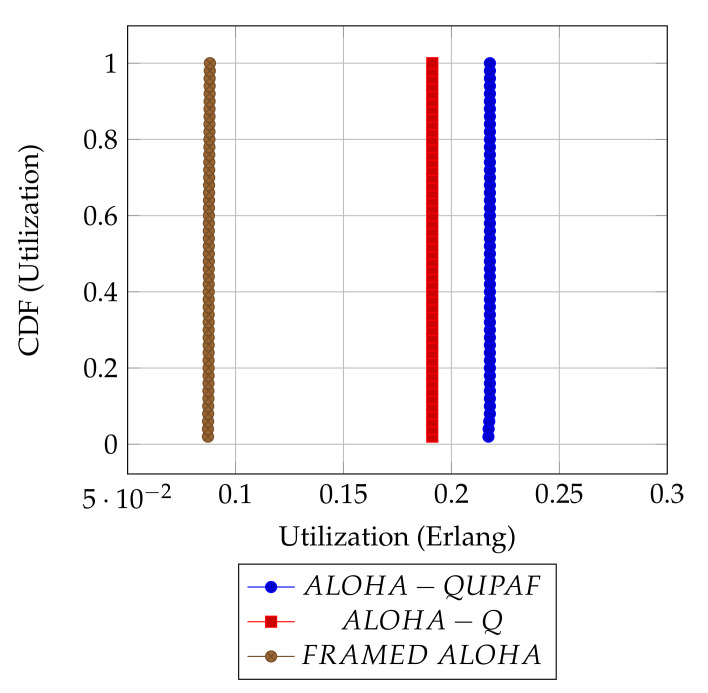
$K_{\tau }>1$: 4 hops utilisation performance comparison.

**Figure 6:**
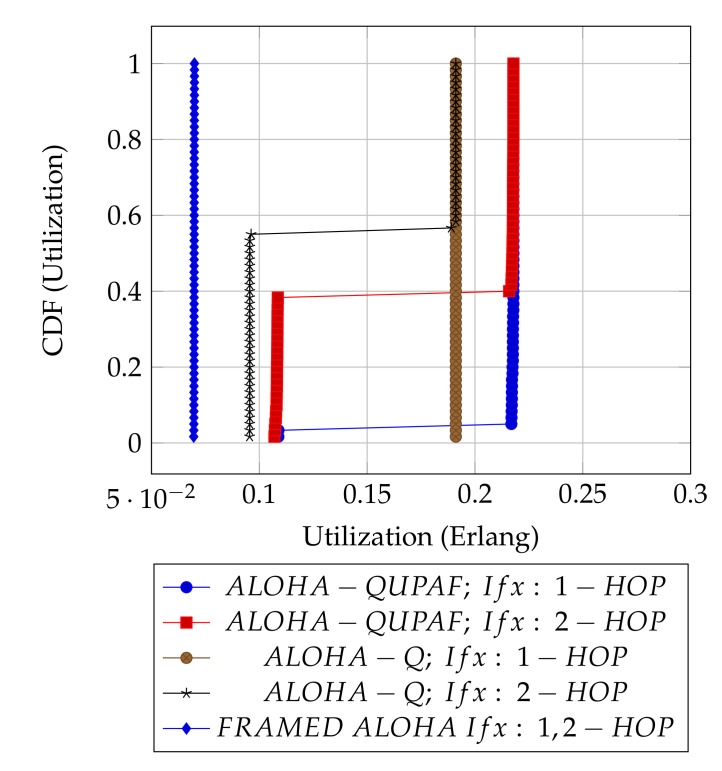
$K_{\tau }>1$: 8 hops utilisation performance comparison.

Figure 5 and Figure 6 are the results obtained when the network was simulated on four and eight hop networks, respectively. The figures compare the performance of ALOHA-QUPAF with ALOHA-Q and framed ALOHA. This comparison is particularly important as the protocols share similar reception conditions in the Kτ>1 scenario; transmission and reception occur in the same slot (Figure 1). Evidently, in this setup, both ALOHA-QUPAF and ALOHA-Q are dramatically affected as the network size increases (four hops to eight hops). The maximum utilisations of ALOHA-QUPAF (0.217 Erlang) and ALOHA-Q (0.191 Erlang) are both sharply halved for about 40% and 58% of the simulated cases, respectively. This performance drop can be explained by the presence of the hidden node phenomenon [38,39]. This is simply the situation whereby a particular communication between any two nodes is interfered by another transmission within range of the receiver.

**Figure 7:**
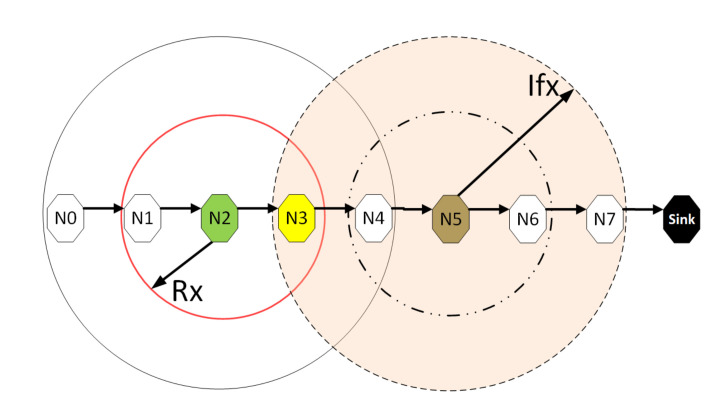
The hidden node problem.

Figure 7 depicts the hidden node problem in an eight hop chain network, in a situation whereby both N2 and N5 share the same transmission slots; thus, transmission from N2 to N3 will be periodically interfered by N5 transmitting to N6, as packets are relayed along the chain. The effect of the hidden node problem as the reason for the performance degradation is confirmed by the agreement shown in the simulation results obtained when the interference range (*Ifx*) is reduced from two hops to one hop in the eight hop chain (Figure 6) with the results in the four hops network (Figure 5). This is because, in a two hop interference range model, a four hop range chain network is of insufficient length for the issue to manifest. Mitigating the hidden node issue is a subject of further work. Another important metric worth mentioning is the end-to-end delay; however, it is not presented here, since ALOHA-QUPAF does not implement packet retransmissions. Therefore, neglecting any processing and queuing delays in the nodes, the E2E delay is fixed as a function of the number of hops in the network. The simulations show that ALOHA-QUPAF achieves 0.124 Erlangs at its worst and 0.248 Erlangs at its best, outperforming both ALOHA-Q (0.19 Erlangs best) and framed ALOHA (0.069 Erlangs) respectively by at least 13% and 148% in all simulated scenarios.

**Figure 8:**
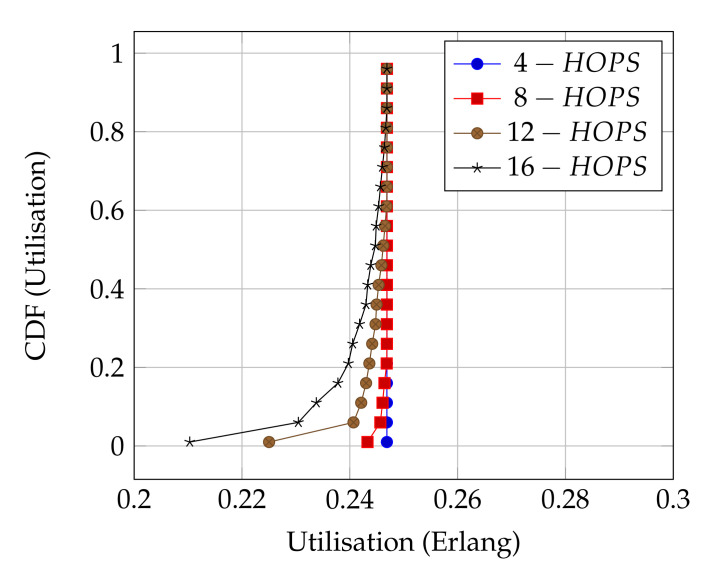
ALOHA-QUPAF utilisation for 4, 8, 12 and 16 hops networks using the proposed slot structure.

Figure 8 presents the performance of ALOHA-QUPAF with our proposed slot structure (Figure 2) in the Kτ<1 scenario. To demonstrate how the ALOHA-QUPAF protocol is affected by the network length, we extend the range to 16 hops and evaluate its performance. The results show a subtle drop in the overall performance from four to 16 hops. The decrease in performance is attributable to the increase in the hidden node spots (bottlenecks points) and the time needed for the protocol to find a collision-free schedule as the network size increases. Each time a node switches to a different transmission slot, this will have a ripple effect across the neighbouring nodes, causing others to potentially switch slots as well, essentially resetting the process. Despite a lack of an explicit acknowledgement signal, the protocol demonstrates significant performance improvement with more than 90% of cases achieving 0.24 Erlangs for networks in the 4–12 hop range and 80% for the 16 hop range.

## 6. Conclusions

In this work, we present a simple slot structure based on the relationship between packet transmission duration and propagation delays in conjunction with two stage reinforcement learning techniques to develop a novel MAC protocol (ALOHA-QUPAF) that can achieve near channel capacity utilisation in a UASN chain topology. Our solution addresses the excessive overhead required in slot structures used by typical slotted/framed protocols. Incorporating a Q-learning in the protocol makes it robust against network and channel changes due to the high dynamic underwater environment. Furthermore, one of the primary goals is for the protocol to be distributed, adaptive, simple and computationally inexpensive so that it is suitable for use in inexpensive and low capacity modems.

To implement our solution, firstly, we analyse the slot structure using an intuitive diagrammatic representation to map the achievable channel utilisation levels. We then reformulate a Q-learning routine that exploits an implicit feedback signal to negatively reinforce and isolate reception slots in the slot selection phase. Secondly, by averaging the packet flow rate, we are able to generate a distribution for belief states that control and consolidate the choice of transmission slot to achieve overall network wide packet flow. We finally evaluate and demonstrate that ALOHA-QUPAF significantly outperforms the comparable protocols with similar Q-learning and slotting concepts.

## Figures and Tables

**Table 1:** Table of mathematical terms.

Entry	Description
N	Number of nodes
$S_{L}$	Number of slots per frame
$N_{opt}$	Optimum number of slots per frame
U	Channel utilisation
$\tau_{d}$	Data packet duration
$\tau_{A}$	ACK packet duration
$\tau_{g}$	Guard duration
Kτ	Ratio of $\tau_{d}$-to-$\tau_{pg}$
$S^{a}$	Slot size with ACK
$S^{n}$	Slot size without ACK
α and γ	Learning rates
λ	Optimisation scale
*fl* ${}_{\tau }$	Packet flow average

**Table 2:** Possible slot permutations.

S/N	Slot Sequence			
	SEQ_0XXX	SEQ_1XXX	SEQ_2XXX	SEQ_3XXX
0	[ 0 0 0 0 ]	[ 1 0 0 0 ]	[ 2 0 0 0 ]	[ 3 0 0 0 ]
1	[ 0 0 0 1 ]	[ 1 0 0 1 ]	[ 2 0 0 1 ]	[ 3 0 0 1 ]
…	[ … ]	[ … ]	[ … ]	[ … ]
…	[ … ]	[ … ]	[ … ]	[ … ]
62	[ 0 3 3 2 ]	[ 1 3 3 2 ]	[ 2 3 3 2 ]	[ 3 3 3 2 ]
63	[ 0 3 3 3 ]	[ 1 3 3 3 ]	[ 2 3 3 3 ]	[ 3 3 3 3 ]

**Table 3:** Summary of utilisation levels.

Level	Proportions (%)				
	SEQ_0XXX	SEQ_1XXX	SEQ_2XXX	SEQ_3XXX	Overall
Worst case (0 E)	45.3	50.0	53.1	43.8	48.1
Intermediate (0.03 E–0.1 E)	9.4	10.4	6.3	10.9	9.4
Half (0.125 E)	21.9	15.6	17.2	21.8	19.1
Maximum (0.25 E)	23.4	23.4	23.4	23.4	23.4

**Table 4:** Example of Q-value update in ALOHA-Q.

Frame/Q-Values	Q[0]	Q[1]	Q[2]	Q[3]
FRAME 0	0	0	0	0
FRAME 1	0	0	−0.1	0
FRAME 2	0	0.1	−0.1	0
FRAME 3	0	0.1900	−0.1	0
FRAME 4	0	0.2710	−0.1	0
…	…	…	…	…
FRAME 20	…	0.8499	−0.1	0

**Table 5:** Simulation parameters.

Parameter	Value
Transmission/Reception Data Rate	640 bps
Data Packet Size	632 bits
ACK Packet Size	16 bits
Slot Size	640 bits
Slots per Frame	4
Reception Range	200 m
ψ	±1
α	0.1
λ	±0.1
γ	0.2
1 hop Propagation Delay (Relative to Packet Size)	1 s

**Figure A1:**
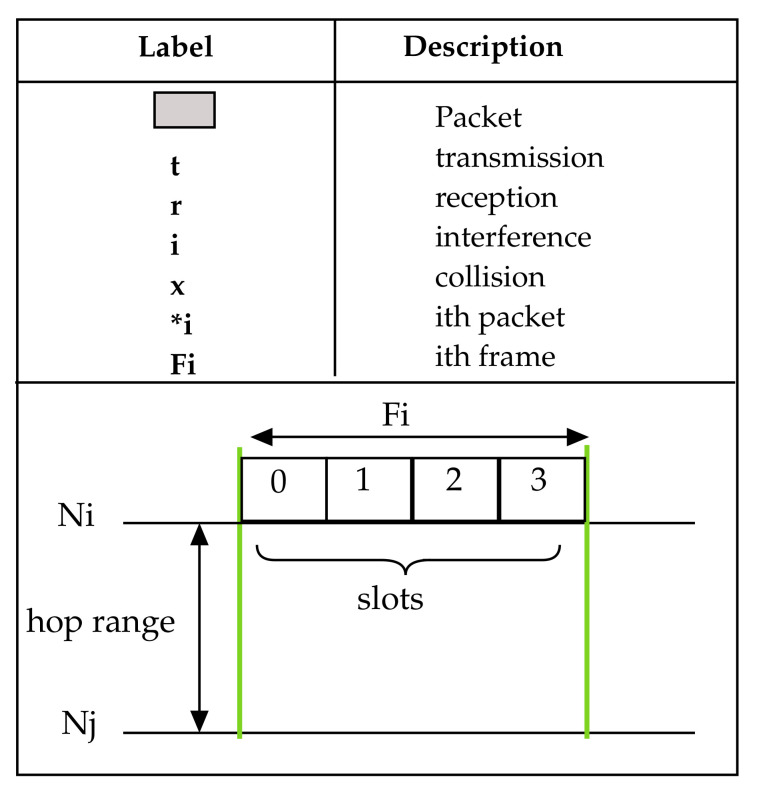
Legend for packet labels and illustrations.

## Data Availability

Not applicable.
